# Venetoclax durable response in adult relapsed/refractory Philadelphia-negative acute lymphoblastic leukemia with *JAK/STAT* pathway alterations

**DOI:** 10.3389/fcell.2023.1165308

**Published:** 2023-05-23

**Authors:** Anna Ferrari, Delia Cangini, Andrea Ghelli Luserna di Rorà, Annalisa Condorelli, Marta Pugliese, Giovanni Schininà, Sebastiano Cosentino, Eugenio Fonzi, Chiara Domizio, Giorgia Simonetti, Salvatore Leotta, Giuseppe Milone, Giovanni Martinelli

**Affiliations:** ^1^ Biosciences Laboratory, IRCCS Istituto Romagnolo per lo Studio dei Tumori (IRST) “Dino Amadori”, Meldola, Italy; ^2^ Hematology Unit, IRCCS Istituto Romagnolo per lo Studio dei Tumori (IRST) “Dino Amadori”, Meldola, Italy; ^3^ Fondazione Pisana per Scienza ONLUS, Pisa, Italy; ^4^ Divisione di Ematologia con Trapianto Emopoietico—Azienda Ospedaliera Universitaria Policlinico “G. Rodolico- San Marco”, Catania, Italy; ^5^ Divisione di Medicina Nucleare, Ospedale Cannizzaro, Catania, Italy; ^6^ Unit of Biostatistics and Clinical Trials, IRCCS Istituto Romagnolo per lo Studio dei Tumori (IRST) “Dino Amadori”, Meldola, Italy; ^7^ Department of Life Sciences and Biotechnology, Ferrara University, Ferrara, Italy; ^8^ Scientific Directorate, IRCCS Istituto Romagnolo per lo Studio dei Tumori (IRST) “Dino Amadori”, Meldola, Italy

**Keywords:** acute lymphoblastic leukemia, venetoclax (BCL-2 inhibitor), Philadelphia-negative cells, *JAK/STAT*, extramedullary

## Abstract

High-risk relapsed/refractory adult Philadelphia-negative (Ph−) B-cell acute lymphoblastic leukemia (B-ALL) is a great challenge due to limited possibilities to achieve and maintain a complete response. This also applies to cases with extramedullary (EM) involvement that have poor outcomes and no accepted standard therapeutic approaches. The incidence of EM localization in relapsed/refractory B-ALL is poorly investigated: data on patients treated with blinatumomab reported a 40% rate. Some responses were reported in EM patients with relapsed/refractory B-ALL treated with inotuzumab ozogamicin or CAR-T. However, molecular mechanisms of response or refractoriness are usually investigated neither at the medullary nor at EM sites. In the complex scenario of pluri-relapsed/refractory B-ALL patients, new target therapies are needed. Our analysis started with the case of an adult pluri-relapsed Ph− B-ALL patient, poorly sensitive to inotuzumab ozogamicin, donor lymphocyte infusions, and blinatumomab in EM disease, who achieved a durable/complete response after treatment with the BCL2-inhibitor venetoclax. The molecular characterization of medullary and EM samples revealed a tyrosine kinase domain *JAK1* mutation in the bone marrow and EM samples at relapse. By comparing the expression level of *BCL2*- and JAK/STAT pathway-related genes between the patient samples, 136 adult *JAK1* ^wt^ B-ALL, and 15 healthy controls, we identified differentially expressed genes, including *LIFR*, *MTOR*, *SOCS1/2,* and *BCL2/BCL2L1*, that are variably modulated at diverse time points and might explain the prolonged response to venetoclax (particularly in the EM site, which was only partially affected by previous therapies). Our results suggest that the deep molecular characterization of both medullary and EM samples is fundamental to identifying effective and personalized targeted therapies.

## Introduction

Acute lymphoblastic leukemia (ALL) is a hematological neoplasm characterized by the uncontrolled proliferation of undifferentiated lymphoid cells that can invade the bone marrow, blood, and extramedullary (EM) sites. As opposed to pediatrics, ALL incidence in the overall cancer types in adults is less (https://www.cancerresearchuk.org). Despite impressive advancements in the treatment of adult Philadelphia-negative (Ph−) B-ALL, patients’ survival rates remain dismal (Brown et al., 2021). Indeed, less than 10% of relapsed/refractory (R/R) B-ALL patients are long-term survivors (Hoelzer et al., 2016), thus urging the need for novel and personalized therapies. Of note, extramedullary relapse incidence is unknown, but it was recently reported as a common event (up to 40%) in R/R ALL patients, following exposure to blinatumomab (Aldoss et al., 2017; Lau et al., 2021). In 84% of R/R B-ALL patients with extramedullary disease, inotuzumab ozogamicin seems to be effective as a debulking strategy (Kayser et al., 2022). Moreover, CAR-T cell therapies achieved encouraging results in the eradication of EM B-ALL (Holland et al., 2022).

The BCL2 inhibitor venetoclax has shown impressive activity in hematological malignancies, including pediatric and adult acute leukemia, both at the preclinical (Pan et al., 2014; Frismantas et al., 2017; Diaz-Flores et al., 2019) and clinical levels. It is currently used for the treatment of adult patients with chronic lymphocytic leukemia (CLL) (Roberts A. W. et al., 2016), and it has been approved by the FDA for acute myeloid leukemia ineligible for intensive chemotherapy in association with hypomethylating agents or low-dose cytarabine (DiNardo et al., 2018, 2019; Lasica and Anderson, 2021). Different studies showed the efficacy of venetoclax in the treatment of T-ALL (Peirs et al., 2014; Rahmat et al., 2018; Farhadfar et al., 2021), including early T-cell progenitor (ETP) ALL (Ni Chonghaile et al., 2014) and B-ALL (Hohtari et al., 2022; Zhang et al., 2022), especially those with *MLL* rearrangements (Benito et al., 2015; Richter-Carpentier et al., 2022). Recently, a phase I clinical trial evaluated its safety and efficacy in combination with low-intensity chemotherapy and navitoclax (a dual BCL2/Bcl-xl inhibitor) in R/R B/T-ALL and lymphoblastic lymphoma. In the study, complete remission (CR) was reached in 59.6% of patients and minimal residual disease (MRD) negativity in 34% of patients (#NCT03181126) (Pullarkat et al., 2021). A study in R/R T-ALL showed clinical efficacy in terms of the bone marrow (BM) response rate in B/T-ALL patients treated with venetoclax-based combination regimens (Richard-Carpentier et al., 2020). Treatment with venetoclax, ponatinib, and dexamethasone (VPD) in Ph-positive (Ph+) ALL patients (T315I-mutated) also showed promising clinical results (Wang et al., 2022). Recent comprehensive reviews reported up-to-date clinical experiences with venetoclax in lymphoid malignancies (Seymour, 2022) and, in particular, in ALL (Aumann et al., 2022) with both chemo and chemo-free regimens. However, different from the CLL experience, in which some resistance mechanisms (e.g., *TP53* aberrations and some *BCL2*, *NOTCH1*, and *BRAF* mutations) have been described, in ALL, the genetic determinants of treatment response and failure have been poorly characterized (Gibson et al., 2022). Although data support the clinical use of venetoclax for R/R ALL patients, the identification of novel predictive markers of response in this high-risk population, affected by both medullary and extramedullary disease, is imperative.

## Methods

### Sample collection

Total mononuclear cells (MNCs) were isolated from peripheral blood (PB) or bone marrow (BM) samples of 105 Triple Negative and 31 Ph + B-ALL patients using LymphoSep (Biowest, Nuaillé, France). A total of 15 samples from healthy subjects were processed, including hematopoietic stem progenitor cells (CD34^+^) from bone marrow specimens (n = 3), bone marrow mononuclear cell samples (n = 3) from STEMCELL Technologies (Vancouver, Canada), PB MNC samples (n = 5), and cord blood samples (n = 4). CD34^+^ cells were enriched from cord blood samples by immunomagnetic separation (CD34 MicroBead Kit, Miltenyi Biotec, Bergisch Gladbach, Germany).

### DNA and RNA extraction

DNA and RNA were extracted from mononuclear cell lysates (buffer RLT, QIAGEN, Hilden, Germany, plus 1% 2β-mercaptoethanol, Life Technologies, Carlsbad, United States) using the AllPrep Mini or Micro Kit (QIAGEN), according to the manufacturer’s instructions. DNA was extracted from formalin-fixed paraffin-embedded (FFPE) inguinal lymph node cells using the Maxwell RSC DNA FFPE kit (Promega, Madison, United States).

### Sanger sequencing

PCR amplifications for the identification of the V651 *JAK1* mutation were performed using the FastStar High Fidelity PCR System (Roche, Mannheim, Germany), as suggested in the datasheet and using 2.5 µL of the 10 µM diluted primer (JAK1 exon14 5′-3′ primers: Forward- GAG​CTT​TCC​TGG​GTC​CAC​T and Reverse- CCA​CCC​CTT​TGA​AAG​AGA​ACA; 61°C annealing temperature) in a 25 µL of the final volume PCR reaction. For each reaction, we used 50 ng of patient sample DNA. PCR products were purified (QIAquick PCR Purification Kit; QIAGEN), and Sanger sequencing was performed using the BigDye Terminator V.3.1 Sequencing Kit (Applied Biosystems, Foster City, California, United States).

### Targeted RNA sequencing

Starting from 40 ng of RNA or 100 ng of FFPE RNA, libraries were prepared using the TruSight RNA Pan-Cancer panel kit (Illumina, San Diego, California, United States), a 1,385 gene panel, following the manufacturer’s protocol. Sequencing was performed using the Illumina MiSeq instrument. In libraries that passed quality checks, paired-end RNA sequencing was performed (Reagent Kit v3 -150 cycles, MiSeq, Illumina).

### Transcriptomic data analyses

Raw sequencing data were converted to FASTQ file format and analyzed for fusion detection combining FusionCatcher, STAR-Fusion, and two BaseSpace applications [RNA-Seq Alignment v.1.1.0 and TopHat Alignment v.1.0.0; Illumina]. The reference “*Homo sapiens* UCSC hg19” (RefSeq and Gencode gene annotations) was used for all the aligners. We retained fusions detected by at least three tools, and we introduced further criteria to retain or reject fusions detected by one or two tools [see PCT application No. PCT/EP 2021/065692 (10/06/2021): Method to identify linked genetic fusions].

For single-nucleotide variations, we analyzed the RNA-Seq Alignment v.1.1.0 “FilteredSmallVariants” output. We kept variants that had a frequency population less than 0.01 into at least one public database of human polymorphisms used in the software [esp5400 (https://evs.gs.washington.edu/EVS/); ExAC (http://exac.broadinstitute.org/); and GnomAD (https://gnomad.broadinstitute.org/)]; we discarded synonymous, 3′/5′ UTR, intronic, and intergenic variants; we retained variants annotated by ClinVar as pathogenic or likely pathogenic; and we also rejected benign/likely benign variants (https://www.ncbi.nlm.nih.gov/clinvar/).

Paired-end reads were trimmed with Trimmomatic (v0.39) (Bolger et al., 2014) using the following arguments: ILLUMINACLIP:TruSeq3-PE.fa:2:30:10 LEADING:3 TRAILING:3 SLIDINGWINDOW:4:15 MINLEN:36. Trimmed reads were pseudo-aligned to a reference transcriptome based on GRCh38 (https://github.com/pachterlab/kallisto-transcriptome-indices/releases/download/ensembl-96/homo_sapiens.tar.gz) with Kallisto (v0.46.2) (Bray et al., 2016), setting 100 bootstraps. The resulting transcripts per million (TPM) were normalized and aggregated to gene level with sleuth (v0.30.0) (which_df = ‘obs_norm’ and which_units = ‘tpm’) (Pimentel et al., 2017).

TPM normalization was performed separately for each group of samples, adding one of the three time points. Downstream analyses were conducted on *JAK/STAT* pathway genes from the KEGG database and *BCL2*-related genes present in the 1,385 TruSight RNA Pan-Cancer Panel gene list (Supplementary Table S1). *BCL2*-related genes were selected from the STRING database (STRING database v. 11.5; https://string-db.org) by applying the standard settings with the exception of the confidence set to a value greater than 0.700 and a maximum number of 20 interactors.

### Statistical analyses

The normalized TPM values were log2-transformed (log2 (x+1)) and used to compute z-scores. The Z-score was considered significant at |z|>1.96. All computations and plots were performed with Python (v3.9.1) or R (v4.0.3).

For protein–protein interaction (PPI) analysis, genes were selected based on the z-score significance (|z|>1.96). PPI was performed by STRING. The minimum required interaction score has been set to ≥0.700 (high confidence value ≥0.700 and very high confidence value ≥ 0.900).

## Results

### Venetoclax-induced CR in a relapsed/refractory Ph− B-ALL patient

**FIGURE 1:**
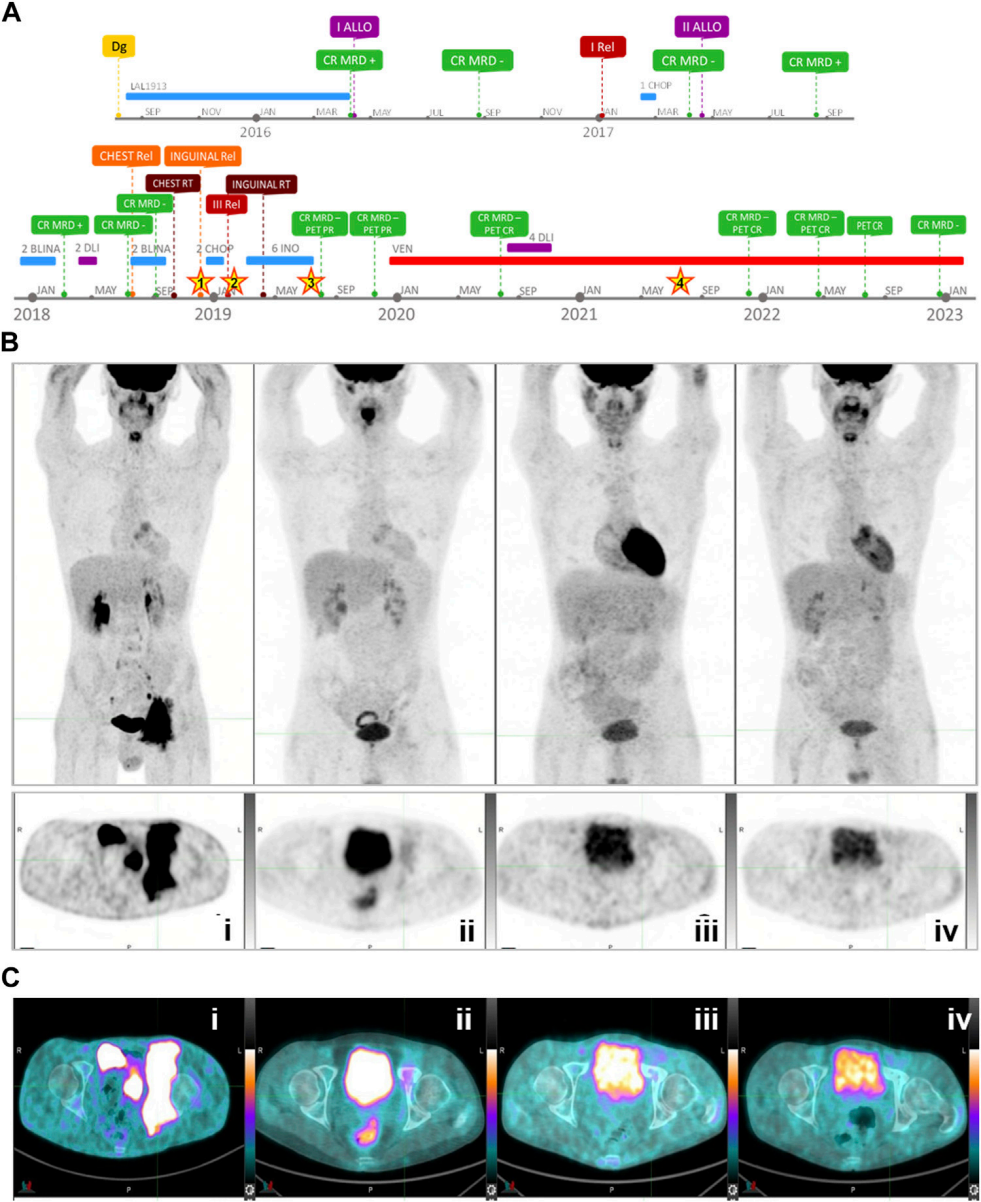
**(A)** Timeline of patient’s leukemia medical history (Dg, diagnosis; ALLO, allogeneic transplantation; MRD, minimal residual disease; CR, complete response; Rel, relapse; DLI, donor lymphocyte infusion; RT, radiotherapy; BLINA, blinatunomab; INO, inotuzomab ozogamicin; PET PR, partial response; VEN, venetoclax; “Stars” indicate the four analyzed time points (1: extramedullary relapse #1—Dec/2018; 2: third relapse #2—Jan/2019; 3: post-inotuzomab ozogamicin hematological remission #4—Jun/2019; 4: post-venetoclax remission—Jul/2021). **(B)** Whole-body (upper panel) and axial scan (lower panel) 18F-FDG PET at four time points: at relapse, in December 2018 (i); after six cycles of inotuzumab ozogamicin followed by radiotherapy, in November 2019 (ii); after 6 months of treatment with venetoclax, in July 2020 (iii), and after 27 months of treatment with venetoclax and four DLIs, in April 2022 (iv). **(C)** 18F-FDG PET images (axial scan) at four time points showing **i)** inguinal pelvic mass at relapse - SUV 9.5 (December 2018); **ii)** partial resolution—SUV 2.9/Deuville 3 (November 2019) reached after treatment with inotuzumab ozogamicin and radiotherapy; **iii)** complete resolution—SUV 1.8 (July 2020—Deuville 1) after 6 months of venetoclax, and **iv)** after 27 months of continuative treatment with venetoclax and four DLIs—SUV 1.4 (April 2022—Deuville 1).

A 28-year-old male patient with no past medical history presented with fatigue in August 2015. The blood count revealed anemia, thrombocytopenia, and leukocytosis (Hb 9.2 g/dL, PLT 120.000/mmc, and WBC 6.500/mmc with an inversion of the formula). Bone marrow evaluation showed a hypercellular marrow with 92% of blast cells. The blast population expressed immature B-cell markers. It was characterized by the presence of nuclear terminal deoxynucleotidyltransferase (TdT), along with CD19^+^, HLA-DR^+^, CD33^+^, CD38^+^, CD71^+^, CD79a^+^, CD22^+^, CD450^+−^, CD10^+^, and CD34^+^ surface phenotypes. This was consistent with the diagnosis of B-ALL. Cytogenetic analysis revealed 46,XY (13),46,XY,del (12) (p11p13) (4); 49,XY,+X,del (12) (p11p13),+13,+21 (3) karyotype. *BCR-ABL1* (Ph+), *TCF3-PBX1*, and *KMT2A-AFF1* transcripts were negative, thus defining the case as a triple-negative (TN) B-ALL. Diagnostic and therapeutic lumbar punctures always resulted in negative localization of disease. Figure 1A shows the timeline of the medical history. The patient received multi-agent pediatric-like chemotherapy for six cycles and achieved complete remission (CR) with a positive MRD. In April 2016, the patient underwent allogeneic hematopoietic stem cell transplantation (allo-HSCT) from a 10/10 matched unrelated donor (MUD). The conditioning was myeloablative with cyclophosphamide 120 mg/kg, total body irradiation (TBI) 12 Gy, and rabbit-thymoglobuline (ATG - Genzyme) 5 mg/kg. Moreover, six months after allo-HSCT, a bone marrow evaluation revealed 10% blast cells by flow cytometry. After a re-induction with vincristine, idarubicine, and cyclophosphamide, the patient reached CR with persistent MRD and, in April 2017, underwent a second allo-HSCT from a 9/10 MUD. The second transplantation was conditioned with a reduced intensity regimen (RIC) based on fludarabine 200 mg/m2, busulfan 6.8 mg/kg, and rabbit-ATG (Genzyme) 5 mg/kg. After initial CR with MRD-negativity, 5 months after allo-HSCT, a bone marrow evaluation revealed 20% of blast cells. The patient then received two cycles of blinatumomab, followed by two donor lymphocyte infusions (DLIs) from the donor of the second transplantation. The dosage of CD3^+^ cells administered was 3.6 and 7 × 10^6^/Kg, respectively. The patient achieved a CR with MRD negativity in the bone marrow. During treatment with blinatumomab, the patient developed an isolated EM disease relapse as a tumefaction on the chest. Fine-needle aspiration of the mass demonstrated the presence of blast cells expressing the CD19 marker. The patient underwent two more cycles of blinatumomab, but the tumefaction kept increasing during treatment. Local radiotherapy (2,600 Gy) was administered to the mass, achieving total regression. The mass appeared after the second DLI and regressed only after irradiation, making it difficult to establish a well-defined role for the adoptive immunotherapy given by DLI in this clinical outcome. The bone marrow evaluation performed after four cycles of blinatumomab confirmed MRD negativity and 100% donor chimerism. However, after the fourth cycle of blinatumomab, in December 2018, the patient presented an enlarged inguinal lymph node (CD19^‒^, CD22^+^) and BM relapse with 50% of blasts. The 18-FDG PET showed intense uptake of the pelvic and inguinal nodes (SUV 9.5; Figures 1B(i)–C(i)).

Between December 2018 and April 2019, the patient was treated with inotuzumab ozogamicin for six cycles, followed by local inguinal radiotherapy (2,500 Gy), achieving CR with MRD negativity in the BM and partial remission of the inguinal-pelvic mass. Partial remission was confirmed by PET in July and November 2019 (SUV 2.9 and 2.7, respectively, in July 2019; 1.5 and 2.1, respectively, in November 2019; Figures 1B(ii)–C(ii)). The treatment with chimeric antigen receptor T cells (CAR-T) for an adult patient aged over 25 was off-label, and there were no compassionate-use programs at the time in Italy. In December 2019, in consideration of the emerging data supporting the use of venetoclax in ALL and the lack of valid therapeutic alternatives, salvage therapy with venetoclax 400 mg once daily was started. A PET scan performed in July 2020 showed no evidence of disease (Figures 1B(iii)–C(iii)). From July to October 2020, during the continuative treatment with venetoclax, the patient also received four escalated-dose DLI from the donor of the second transplantation (infused CD3^+^ cells were, respectively: 5 × 10^6^/Kg, 2,5 × 10^7^/Kg, 5 × 10^7^/Kg, and 5 × 10^7^/Kg). Two subsequent PETs in November 2021 and April 2022 showed complete resolution of pelvic masses, and MRD negativity was maintained in the BM (Figures 1B(iv)–C(iv)). Continuative therapy with venetoclax is well-tolerated, and no adverse events have occurred to date. The most recent evaluations performed in July 2022 by PET and in December 2022 by bone marrow aspiration confirmed the CR.

### *JAK1* mutations at disease relapse

**FIGURE 2:**
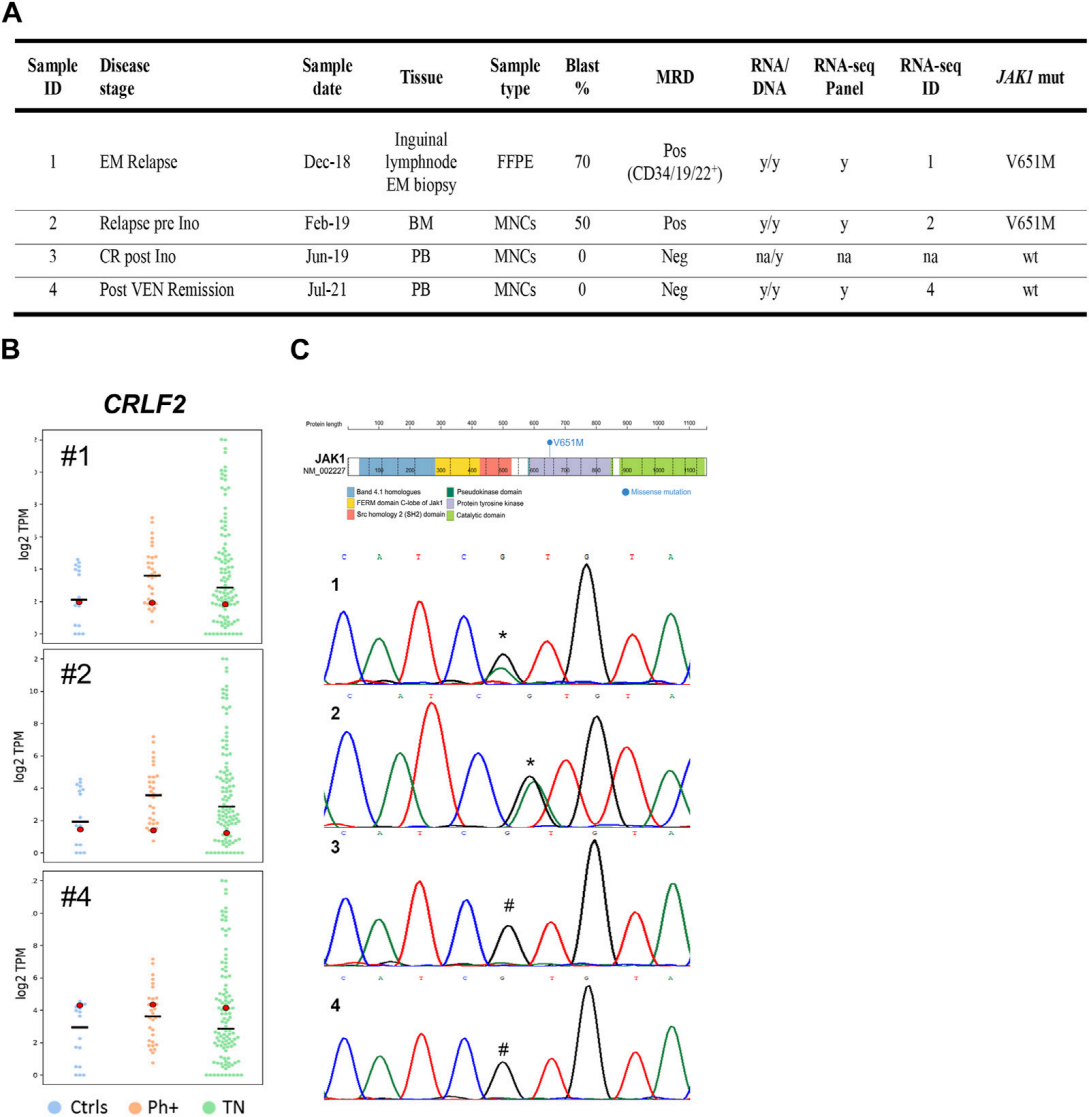
**(A)** Characteristics and analyses of patient’s sample at each time point. BM, bone marrow; EM, extra-medullary; FFPE, formalin-fixed paraffin-embedded; Ino, inotuzumab ozogamicin; MNCs, mononuclear cells; MRD, minimal residual disease; mut, mutation; na, not available; Neg, negative; PB, peripheral blood; Pos, positive;VEN, venetoclax; wt, wild type; and y, yes. **(B)** CRLF2 expression analysis in the patients’ samples and comparison groups. For the *CRLF2* gene, swarm plots of log-transformed TPM are displayed. Each plot is a combination of one group of samples with one time point sample. The time point sample is highlighted with a bigger dot, and its color changes, depending on its z-score value (green if |z|>1.96; otherwise, red). The horizontal black lines mark the median. Ctrls, healthy donor group (n = 15); Ph+, *BCR-ABL1*-positive group (n = 31); TN, triple-negative group (n = 105). **(C)** *JAK1* mutation in the patient’s sample. JAK1 protein diagram with the site of the V651M somatic variant (NM_002227:exon14:c.G1951A:p.V651M; Human hg19) within the tyrosine kinase domain and DNA Sanger sequencing chromatograms of the *JAK1* V651 position at different time points (1: extramedullary relapse- Dec/2018; 2: third relapse—Jan/2019; 3: post-inotuzomab ozogamicin hematological remission—Jun/2019; 4: post-venetoclax remission—Jul/2021). * site of heterozygous mutation (V651M); #V651 wild-type site.

To understand the molecular bases of the durable response to venetoclax in a pluri-relapsed TN patient, we analyzed samples of four patients collected at different time points: EM localization (#1), mononuclear cells from BM (#2) at relapse, peripheral blood cells at remission post-inotuzumab ozogamicin (#3), and post-venetoclax (#4; Figure 2A). We investigated gene fusions, RNA single-nucleotide variations, and gene expression by targeted RNA sequencing on samples #1, #2, and #4. Neither novel nor leukemia-associated fusion genes were found in the analyzed samples. *TP53* and the three most frequently mutated genes in Ph-like B-ALL, *CRLF2*, *IL7R*, and *JAK2* were wild-types (wts) in the analyzed samples (Roberts et al., 2014; Jain et al., 2017; Roberts, 2017; Iacobucci et al., 2021). Moreover, *CRLF2*, which is frequently overexpressed in Ph-like B-ALL (Jain et al., 2017; Iacobucci et al., 2021), was expressed at lower levels in samples #1 and #2 than sample #4, Ph+, and TN B-ALL (Figure 2B, Supplementary Table S2). We identified and confirmed a missense mutation in the tyrosine kinase domain of the *JAK1* gene at the DNA level in samples #1 and #2 (NM_002227:exon14:c.G1951A:p.V651M; hg19), while no *JAK1* mutations were detected in the two remission time points (#3, #4; Figures 2A–C). The functional impact of this alteration has been predicted as highly damaging (score 1.0, PolyPhen2; score 0.97, FATHMM).

### Transcriptional alterations in the BCL2 and JAK/STAT pathways during disease evolution

To investigate the changes occurring in the transcriptional program through the diverse disease stages and tissues in relationship with treatment responses, we analyzed the level of expression of *BCL2*-related genes and JAK/STAT pathway components in the patient’s samples (#1, #2, or #4) and compared it with 136 adult *JAK1* ^wt^ B-ALL cases, including 105 TN and 31 Ph + cases, and 15 healthy controls (Ctrls) (Supplementary Tables S1, S2). We identified several differentially expressed genes (Supplementary Table S3).

**FIGURE 3:**
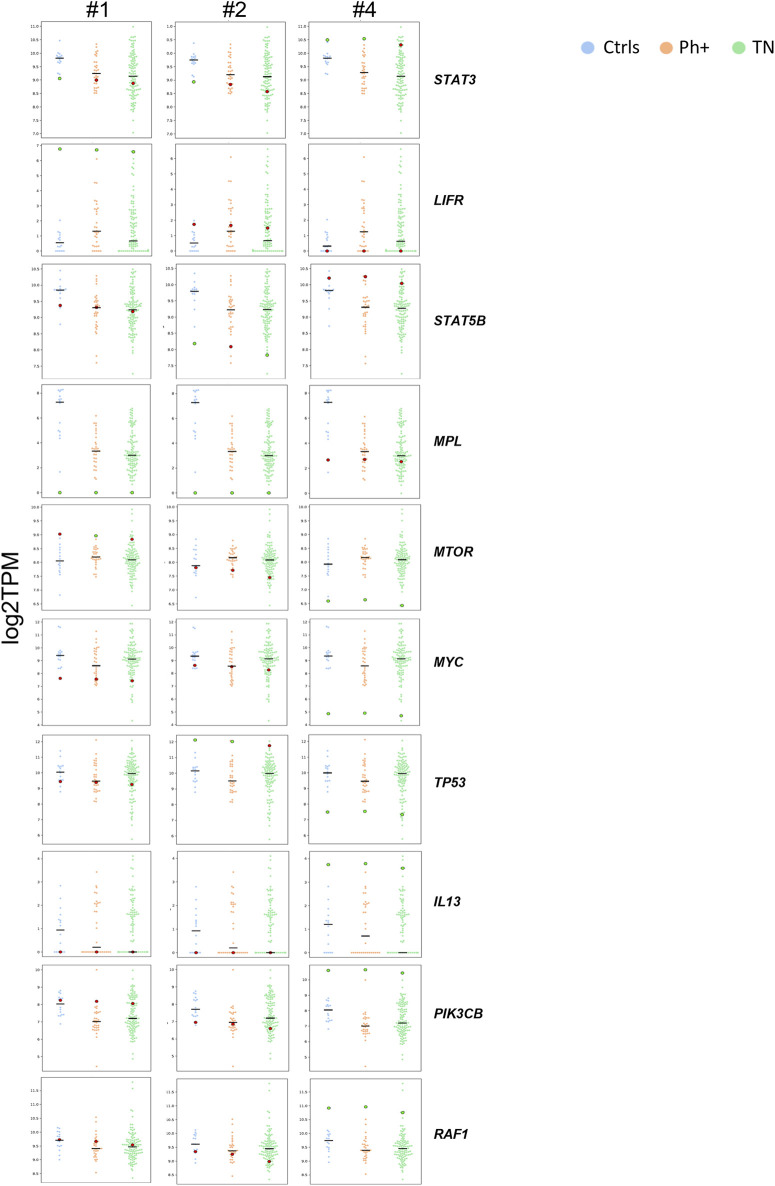
*STAT3, LIFR, STAT5B, MPL, MTOR, MYC, TP53, IL13, PIK3CB*, and differential expression analysis between the patient’s samples and comparison groups. For each gene, multiple swarm plots of log-transformed TPM are displayed. Each plot is a combination of one group of samples with one time point sample. The time point sample is highlighted with a bigger dot, and its color changes, depending on its z-score value (green if |z|>1.96; otherwise, red). The horizontal black lines mark the median. Ctrls, healthy donor group (n = 15); Ph+, *BCR-ABL1*-positive group (n = 31); TN, triple-negative group (n = 105).

The inhibitory factor receptor *LIFR*, which is commonly upregulated in many solid cancers and is involved in the activation of the JAK/signal transducer and activator of transcription 3 (STAT3) pathway, was overexpressed in sample #1 in comparison with all groups (Figure 3). *STAT3* was downregulated in the two relapse samples while being significantly overexpressed in the remission sample, with a higher value than Ctrls. Similarly, our analysis highlighted *STAT5B* downregulation in relapse sample #2 and its elevated expression after venetoclax treatment (sample #4) compared with TN, Ph+, and Ctrl groups, and upregulation of the signaling molecules PIK3CB and RAF1 at remission compared with the other cohorts. Since the data on the transcript level of *STAT3* and *STAT5B* do not allow us to conclude on the activation status of these molecules, we checked for downstream genes. We observed a downregulation of the downstream effector *MTOR* and of the JAK/STAT target *MYC* in remission samples compared to the *JAK1* ^wt^ B-ALL cohorts and Ctrls, suggesting that the pathway is switched off in response to venetoclax. Moreover, *IL13* was significantly upregulated in sample #4 as a potential indication of hematopoietic system restoration (Figure 3). Accordingly, *MPL*, which regulates megakaryopoiesis and platelet production, was expressed in sample #4 at similar levels compared with Ctrls and all B-ALL cohorts while not being detectable in samples #1 and #2 (Figure 3).

**FIGURE 4:**
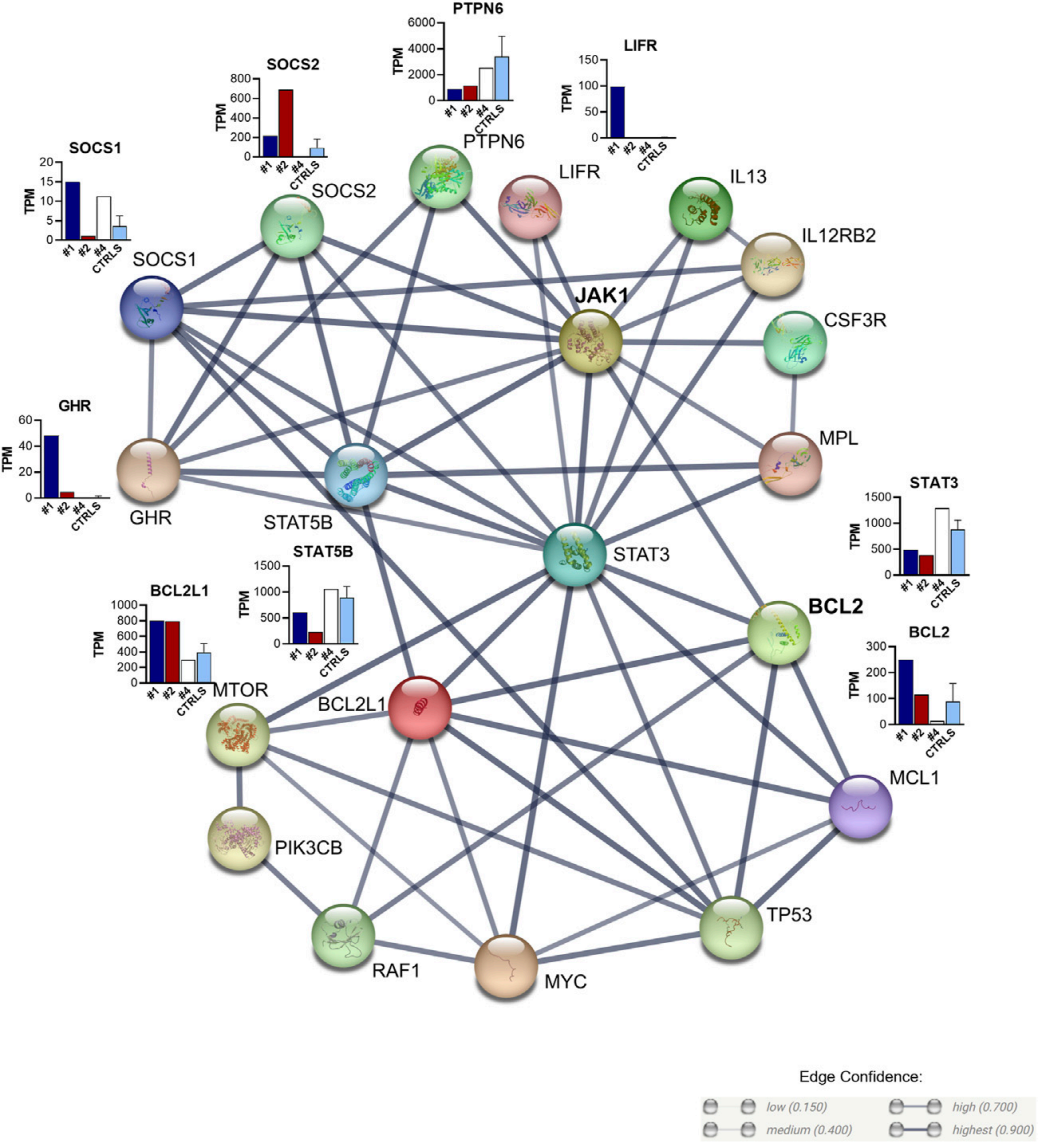
Protein–protein interaction network of the top differentially expressed genes between the patient’s samples and the Ctrl cohort. Edges represent protein–protein associations. Confidence ≥0.700; maximum number of interactors ≤20. Edge confidence: *high (0.700)* and *highest (0.900)* (see https://string-db.org/cgi/network).

To better understand the functional and physical interactions among the mutated *JAK1*, the direct targets of venetoclax, and all genes showing altered expression in the two *JAK1* mutant relapse samples and the post-treatment remission sample, we performed protein–protein interaction (PPI) analysis (STRING database; https://string-db.org). In particular, we compared the transcriptomic profiles of samples #1, #2, and #4 with the Ctrl group, and we performed PPI analysis on the differentially expressed genes with the highest significance (defined as z-score value |z|>1.96; Supplementary Table S3). PPI analysis revealed a tight interconnection between these genes (PPI enrichment *p*-value: 1.0e-16; Figure 4). *JAK1* and *STAT3* were strongly connected with *BCL2* (scores 0.936 and 0.908, respectively; Supplementary Table S4). The connection network underlined the central role of *JAK1*/*STAT3*/*STAT5B* and of the *BCL2*/*BCL2L1* antiapoptotic proteins (that showed variably higher expression levels in relapse samples than the remission and the Ctrls) at the crossroad between the JAK1/STAT altered pathway and downstream cellular and biological processes. Indeed, strong interactions were observed between JAK/STAT and the suppressors of cytokine signaling 1 (*SOCS1*) and *SOCS2*, which were variably upregulated, in particular in the relapse samples; the protein tyrosine phosphatase non-receptor type 6 (*PTPN6*), which was downregulated in the same time points and the growth hormone receptor (*GHR*), which, similar to *LIFR*, are specifically overexpressed in the EM sample.

## Discussion

Few studies have evaluated the efficacy and predictive markers of response to venetoclax in T and, especially, B-ALL so far. Here, we reported the efficacy of a combined treatment comprising inotuzumab ozogamicin, DLI, and venetoclax in a young adult pluri-relapsed B-ALL patient that led to regression of medullary and EM disease. We correlated the molecular background with the treatment response. Inotuzumab ozogamicin induced CR in the BM and partial remission at the EM site, while venetoclax induced a CR of the EM disease and, combined initially with DLI, was able to maintain the CR until the last evaluation, which was performed 3 years after treatment initiation. To date, this is the most durable disease-free survival experienced by the patient.

By analyzing transcriptomic profiles, we detected neither fusion genes nor mutations in driver or key disease-related genes such as *TP53*, *CRLF2*, *IL7R*, and *JAK2.* Moreover, *CRLF2*, which is frequently upregulated in a subgroup of Ph-like cases, was not overexpressed. However, we found a damaging *JAK1* point mutation in both samples #1 and #2, suggesting a potential driver role in relapse. Indeed, the mutation was not detected in remission samples after inotuzomab ozogamicin and venetoclax treatments (#3 and #4). *JAK1* mutations have been described in adult Ph-like cases with non-overexpressed *CRLF2* (Roberts K. G. et al., 2016). Jain N. and others identified a subgroup of adult Ph-like B-ALL patients, lacking *CRLF2* upregulation/alterations (CRLF2^−^ Ph-like) while displaying *JAK1* mutations, similar to our case. This B-ALL subgroup has a dismal prognosis, which is not in any way better than that of CRLF2 + Ph-like ALL (Jain et al., 2017). Among *JAK* genes, *JAK2* is the most frequently mutated in B-ALL, especially in high-risk cases. However, some *JAK1* and *JAK3* mutations have also been reported (Flex et al., 2008; Zhang et al., 2011; Li et al., 2017). The *JAK1* ^V651M^ mutation, which we describe here, has been previously detected in children with Down syndrome ALL (Blink et al., 2009), in T-ALL (Lupardus et al., 2014), and in prostate carcinomas (https://cancer.sanger.ac.uk/cosmic/mutation/). Few pieces of evidence are available in the literature regarding the functional consequences of mutations in the protein tyrosine kinase domain of JAK1 in B-ALL, including *JAK1* ^S646P^ that resulted in a high sensitivity to the JAK1/2 inhibitor ruxolitinib in B-ALL patients and *JAK1* ^V658F^ that led to constitutive JAK1 activation in cell lines (Flex et al., 2008; Jeong et al., 2008; Li et al., 2017). Other *JAK1* single-nucleotide variants were described, also with a gain of function effects in B-ALL (https://www.oncokb.org/gene/JAK1#tab=Biological) (Hammarén et al., 2019), but no data are currently available regarding the V651M variant effects in adult B-ALL.

Although few studies have investigated the efficacy of JAK inhibitors in Ph- ALL, recently Dr. Kołodrubiec and others collected preclinical and clinical pieces of evidence on the efficacy of ruxolitinib as a single agent or in combinations against Ph- ALL (Kołodrubiec et al., 2022). Moreover, additional preclinical studies showed a synergistic effect of ruxolitinib in combination with BCL2 (venetoclax), BCL6 (BI3802, BI3812, and FX1) (Tsuzuki et al., 2023), and LSD1 (GSK2879552) inhibitors in different ALL subtypes (Senkevitch et al., 2018).

Starting from the identified *JAK1* mutation, we compared the expression of different BCL2-related and JAK/STAT pathway genes between our patient and a cohort of adult *JAK1* ^wt^ B-ALL (both TN and Ph + cases) and healthy donors in order to identify markers of response. We identified alterations in several transcripts. *LIFR* and *GHR* were selectively upregulated in the EM sample. The proteins encoded by these genes are involved in the JAK/STAT pathway regulation, particularly in the phosphorylation and activation of JAK1/2 (Smit et al., 1996; Hellgren et al., 2001; Christianson et al., 2021). A prognostic role of LIFR and its ligand (LIF) has been recognized across several human cancers (Christianson et al., 2021), but the importance of this pathway and its co-occurrence with a *JAK1* mutation have never been explored in lymphoid malignancies.

At the crossroads between upstream and downstream players, we observed the alteration of STAT proteins. According to the literature, more than 40 different polypeptide ligands, including cytokines, JAK kinases, and growth factors, are associated with STAT phosphorylation, which, in turn, orchestrates the activation of their effectors (Wang et al., 2020). STAT3 activation is tightly controlled and only occurs within a short window of time during normal immune responses (O’Shea et al., 2013). However, it is well known that dysregulation and constitutive activation of STAT3 are associated with human diseases, including cancer (Bowman et al., 2000; O’Shea et al., 2013).

Indeed, STAT3 regulates the expression of genes involved in cell proliferation, survival, differentiation, migration, angiogenesis, and inflammation, which concur in different ways with malignant transformation and progression (Zhu et al., 2020).

During remission, we observed the expressions of *MPL* and *IL13* that regulate the immune system and were undetectable in both medullary and EM relapses. The MPL protein regulates megakaryopoiesis and platelet formation through the activation of the JAK2 signal and the JAK/STAT pathway (Chou and Mulloy, 2011). In our case, MPL and IL13 restoration in the sample post-venetoclax highlight potential biological roles in response to treatment.

Our clinical observations indicate that the EM disease was partially sensitive to inotuzumab ozogamicin alone; conversely, the patient achieved complete remission after venetoclax treatment combined with DLI.

Historical data show that the results of DLI in treating ALL relapse are disappointing in terms of long-term survival, particularly in cases of overt relapse and high disease burden (Collins et al., 2000; Spyridonidis et al., 2012). This suggests that our patient may have benefited from the combination of DLI and venetoclax rather than DLI alone. In this regard, it is worth noting that venetoclax, in addition to exerting a pro-apoptotic function against malignant cells, is also able to induce anti-leukemic T-cell activation (Lee et al., 2021).

In order to understand the molecular mechanisms that could help explain the therapeutic role of venetoclax, we focused on BCL2-related genes and the JAK/STAT pathway that could collectively contribute to venetoclax long-lasting response in BM and EM. We hypothesize that, in EM disease, *JAK1* lesions and activation of the downstream pathway may, as driver aberrations, lead to the dysregulation of genes involved in cell survival and proliferation (Verhoeven et al., 2020). Therefore, the increased activity of this pathway may be responsible for the responsiveness of the EM disease to BCL2 inhibition. In line with our results, a recent work reported the persistence of a predominant CD22^low^/Bcl-2^high^ leukemic population in pediatric B-ALL patients that poorly responded to inotuzumab ozogamicin, suggesting that they may have benefited from combined treatment with venetoclax (Diaz-Flores et al., 2021).

In conclusion, high-risk-R/R adult TN B-ALL with both medullary and EM involvement is a clinical challenge with few available therapeutic options. Our case suggests that a comprehensive molecular characterization of multiple disease localizations, including both medullary and EM samples, is fundamental in identifying effective and personalized targeted therapies.

## Data Availability

The data presented in the study are deposited in the European Genome-Phenome Archive repository (https://ega-archive.org/), accession number EGAD00001010837.
